# Effects of Hormone, NEFA and SCFA on the Migration of Neutrophils and the Formation of Neutrophil Extracellular Traps in Dairy Cows

**DOI:** 10.3390/ani12091190

**Published:** 2022-05-06

**Authors:** Guanxin Lv, Hai Wang, Xiechen Zhou, Shuai Lian, Jianfa Wang, Rui Wu

**Affiliations:** 1 College of Animal Science and Veterinary Medicine, Heilongjiang Bayi Agricultural University, Daqing 163319, China; lgxarron@foxmail.com (G.L.); whdota@foxmail.com (H.W.); zhouxiechen@163.com (X.Z.); lianshuai@byau.edu.cn (S.L.); wjflw@sina.com (J.W.); 2 Heilongjiang Provincial Key Laboratory of Prevention and Control of Bovine Diseases, Daqing 163319, China

**Keywords:** bovine, neutrophils, hormone, NEFA, SCFA, migration, NETs

## Abstract

**Simple Summary:**

Perinatal dairy cows face the challenge of maintaining the resilience of defense against invading pathogens. During the perinatal period, hormonal or metabolic changes cause the decline of immune function of dairy cows and further lead to varying degrees of immunosuppression. The results of this study indicate that, hormones, nonesterified fatty acids (NEFAs) and short-chain fatty acids (SCFAs) can regulate neutrophil migration and the NETs formation of dairy cows in vitro. These results help to further explain the effects of changes in hormone secretion and metabolites on immunosuppression and the increased risk of disease in perinatal dairy cows.

**Abstract:**

Polymorphonuclear neutrophils (PMN) are the first line of defense against the invasion of foreign pathogenic microorganisms and play an essential role in the immune system of dairy cows. The changes in hormone secretion and metabolites of dairy cows during the perinatal period are the key factors that cause immunosuppression and increased risk of diseases. However, the effects of the hormone, nonesterified fatty acid (NEFA), and short-chain fatty acid (SCFA) on the transmammary epithelial migration of dairy cows and the formation of neutrophil extracellular traps (NETs) have rarely been studied. This study explored the effects of hormones, NEFAs and SCFAs on the neutrophil migration and NETs formation of dairy cows in vitro. It was found that P_4_ and Ac can regulate the transepithelial migration of PMN; SA and Pr can regulate the formation of NETs; E_2_, OA and Bt can regulate PMN transepithelial migration and NET formation. These results help to further explain the effects of changes in hormone secretion and metabolites on immunosuppression and the increased risk of disease in perinatal dairy cows.

## 1. Introduction

Polymorphonuclear neutrophils (PMN) are the first line of defense against the invading pathogens pathogenic microorganisms and play an essential role in the innate immunity of dairy cows [1,2]. During mastitis in a dairy cow, pathogenic microorganisms gather in the acinar lumina and secrete a large number of inflammatory factors. At this time, PMN is recruited to the inflammation site as a response [3]. When PMN cross the mammary epithelium and enter the acinar lumina, they kill invading microorganisms by phagocytosis, the production of reactive oxygen species (ROS), the release of enzymes with bactericidal activity and the formation of extracellular trap (NET) to ensure the health of the cow’s mammary glands [4,5].

Perinatal dairy cows (three weeks before and three weeks after delivery) experience a series of hormonal and metabolic changes [6,7]. In terms of hormone secretion, perinatal dairy cows experience dramatic changes in the secretion levels of 17β-estradiol (E_2_) and progesterone (P_4_) during pregnancy, delivery and lactation. In terms of metabolism, the demand of cows for nutrients in the early stages of lactation exceeds the energy obtained from their feeding, and they experience a transient state of negative energy balance [8]. This state is characterized by hypoglycemia, intense lipid mobilization and increased plasma β-hydroxybutyric acid level [9].

Hormonal or metabolic changes cause the decline of the immune function of dairy cows and further lead to varying degrees of immunosuppression [6,10,11]. During immunosuppression, the speed of PMN chemotaxis and transepithelial migration in dairy cows decreases, and phagocytosis degrades, weakening the mammary immunity, increasing the risk of mastitis and leading to immune impairment [10,12]. E_2_ and P_4_ are the most important reproductive hormones in the developmental and reproductive stages of dairy cows and play an important role in pregnancy and its maintenance [13]. Lamote et al. found that a degraded PMN function in dairy cows after parturition was closely related to E_2_ secretion level [14]. Under intense lipid mobilization, incompletely oxidized nonesterified fatty acid (NEFA), oleic acid (OA), palmitic acid (PA), and stearic acid (SA) significantly inhibited PMN chemotaxis and phagocytosis, with a significant decrease of ROS produced by PMN respiratory burst and a significant increase of the incidence of endometritis [9,15]. Other studies have also shown that NEFA can inhibit PMN respiratory bursts [16]. In addition, short-chain fatty acid (SCFA), acetate (Ac), propionate (Pr) and butyrate (Bt) are produced by the symbiotic microbiota in the gastrointestinal tract. They are the primary energy source for the metabolism of ruminants and satisfy more than 70% of their energy demand [17]. Many studies have shown that SCFAs have a regulatory effect on PMN function. For example, Vinolo et al. found that Ac can increase ROS production of PMN without affecting PMN bactericidal ability; Pr can inhibit phagocytosis and bactericidal ability of PMN [18]. Carretta et al. found that SA can activate PMN, induce Ca^2+^ influx, and promote MAPK phosphorylation [19]. However, the effects of the hormone, NEFA, and SCFA on the transmammary epithelial migration of dairy cows and the formation of NETs have rarely been studied.

This research aims to evaluate the effects of physiological concentration or high-concentration of E_2_, P_4_, OA, PA, SA, Ac, Bt, and Pr in perinatal dairy cows on the transmammary epithelial migration of PMN and the formation of NETs.

## 2. Materials and Methods

### 2.1. Animals

The cows used in the experiments were healthy Holstein cows from a large-scale cattle farm in Anda, Heilongjiang Province. They were in the middle stage of lactation, which could ensure that the obtained cells were in optimal functional condition. All experiments were approved by the Animal Ethics Committee of Heilongjiang Bayi Agricultural University (NO.BYAU20191121).

### 2.2. PMN Isolation

Blood was collected by tail vein puncture under aseptic conditions into a blood collection tube containing sodium citrate, and PMN was isolated using the Solarbio Bovine Peripheral Blood Neutrophil Isolation Kit^®^ (Solarbio, Bejing, China) according to the manufacturer’s instructions. First, 4 mL of Reagent A was added into a 15 mL centrifuge tube, then 2 mL of Reagent C was carefully added onto the surface of Reagent A, and finally, 2 mL of peripheral blood was added into the solution. In order to achieve cell separation, the solution was centrifuged in an Eppendorf high-speed centrifuge at 900× *g* for 30 min (Eppendorf, Hamburg, Germany). The granule cell layer was aspirated, added with RPMI 1640 medium, and washed 3 times by centrifugation at 500× *g* for 5 min. Trypan blue staining was used to determine cell death with the number of trypan-blue-positive and trypan-blue- negative PMN counted by an automatic cell counter, and its concentration was adjusted to 1 × 10^6^ cells/mL.

### 2.3. Cell Culture

Dairy cow mammary epithelial cells (MAC-T) were given by Jilin University. MAC-T cells were cultured with DMEM/F12 (Gibco, Grand Island, NY, USA) in a 37 °C humid atmosphere with 5% CO_2_. This medium was supplemented with 10% fetal bovine serum FBS (Gibco, Grand Island, NY, USA), 100 U/L penicillin and 100 mg/L streptomycin (Solarbio, Bejing, China). The medium was changed once a day, and generation 3 to generation 6 were used for subsequent experiments.

### 2.4. PMN Transepithelial Migration Detection with Transwell

In order to construct the dairy cow mammary epithelium in vitro, MAC-T cells were inoculated into the upper chamber of the Transwell system (3.0 μm, 24-well insert; Corning, Lowell, MA, USA) with a density of 2 × 10^6^ cells/mL and cultured in a 37 °C, 5% CO_2_ wet environment for 3 days. The medium was changed once a day. After more than 90% cell fusion was achieved, the MAC-T cells were washed three times with PBS for later use. PMN (1 × 10^5^ cells/mL) was coincubated with E_2_ (20, 100, and 200 pg/mL), P_4_ (5, 20, and 40 ng/mL), OA (0.065, 0.130, and 0.260 mM), PA (0.05, 0.100, and 0.200 mM), SA (0.02, 0.04, and 0.08 mM), Ac (10, 20, and 30 mM), Bt (4, 8, and 12 mM), and Pr (0.5, 2.5, and 5 mM), respectively, at 37 °C for 30 min to get the experimental group, and it was coincubated with PBS in the same way to get the control group. Then, PMN was added to the upper chamber of the Transwell system, and 400 μL of RPMI 1640 containing 10% FBS was added to the lower chamber. The Transwell system was placed in a 37 °C, 5% CO_2_ incubator for 3 h. The number of PMN migrating to the lower chamber was detected by a flow cytometer (Beckman Coulter, Indianapolis, IN, USA).

### 2.5. Visual Inspection of NETs

The polylysine-treated hepatocytes were placed into a 24-well plate. The PMN were spread on this plate with a density of 2.5 × 10^5^ cells/well. Stimulating reagents of different concentrations were added, as described in the above section “PMN transepithelial migration detection with Transwell”. Then, phorbol ester (PMA) was added, and its final concentration was 100 nmol/L. After that, the hepatocytes were placed into a 37 °C, 5% CO_2_ incubator for 3 h. A total of 4% paraformaldehyde was added and fixed at room temperature for 1 h. PBS-T was added to wash the hepatocytes 3 times. Then, 0.3% Triton Χ-100 was added for permeabilization for 15 min. The hepatocytes were blocked in 10% goat serum for 1 h and then mixed with PBS-buffer-diluted mouse myeloperoxidase (MPO) and rabbit ELA2 (Proteintech Group, Wuhan, China). The solution was incubated overnight in a refrigerator at 4 °C. Then, the hepatocytes were washed 3 times with PBS-T. Alexa Fluor^®^ 488 was added to label goat anti-mouse IgG, and Alexa Fluor^®^ 594 was added to label goat anti-rabbit IgG (ZSGB-BIO, Bejing, China). The hepatocytes were incubated in the dark for 1 h and washed three times. An antifluorescence quenching mounting tablet containing DAPI (Solarbio, Bejing, China) was dropped on the glass slides. The hepatocytes were taken out and placed upside down on the slides. After fixation and mounting, the hepatocytes were observed under a laser confocal microscope (Leica, Wetzlar, Germany). Image J software was used to calculate the total number of cells in a single field of view, and the percentage of the formed NETs was the ratio of the number of NETs formed under the field of view to the total number of cells.

### 2.6. Statistical Analysis

The results are shown in the histogram as the mean ± SD of three independent experiments. A one-way analysis of variance was used for comparison between groups, and Graph pad prism 8.0 was used for data analysis. *p* < 0.05 was considered statistically significant.

## 3. Results

### 3.1. The Effects of E_2_ and P_4_ on PMN Migration and NET Formation

**Figure 1:**
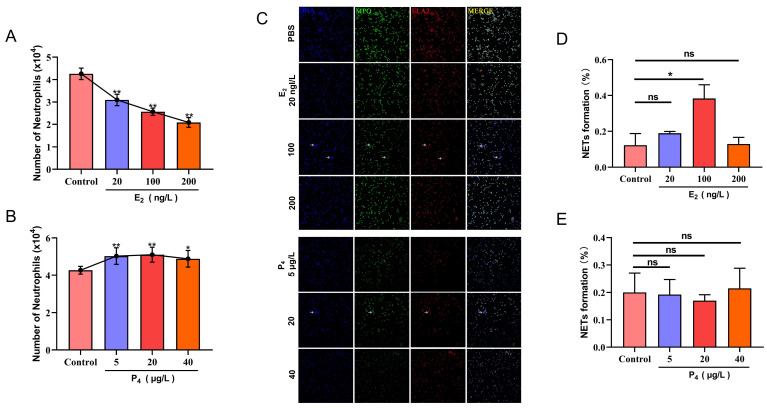
Effects of E_2_ and P_4_ on migration and NET formation of PMN. (**A**) Effects of E_2_ on migration of PMN; (**B**) effects of P_4_ on migration of PMN; (**C**) effects of E_2_ and P_4_ on NET formation of PMN (white arrows: NET); (**D**) effects of E_2_ on the percentage of NET formation; (**E**) effects of P_4_ on the percentage of NET formation. Data are shown with means ± SD (*n* = 3). * *p* < 0.05, ** *p* < 0.01, compared to control group.

Compared with the control group, low, medium, and high concentrations of E_2_ (20, 100, and 200 pg/mL) significant reduced PMN transepithelial migration (*p* < 0.01), and the higher the E_2_ concentration, the more obvious the effect (Figure 1A). P_4_ promoted the PMN transepithelial migration. Compared with the control group, a low concentration (5 ng/mL) and medium concentration (20 ng/mL) of P_4_ significant increased PMN transepithelial migration (*p* < 0.01); a high concentration of P_4_ (40 ng/mL) could also increase PMN transepithelial migration, but the increasing effect tended to weaken (*p* < 0.05) (Figure 1B). Compared with the control group, the medium concentration of E_2_ (100 pg/mL) significantly induced the formation of NETs (*p* < 0.05) (Figure 1C,D); P_4_ had no significant effect on the formation of NETs (Figure 1C,E).

### 3.2. The Effects of OA, PA, and SA on PMN Migration and NET Formation

**Figure 2:**
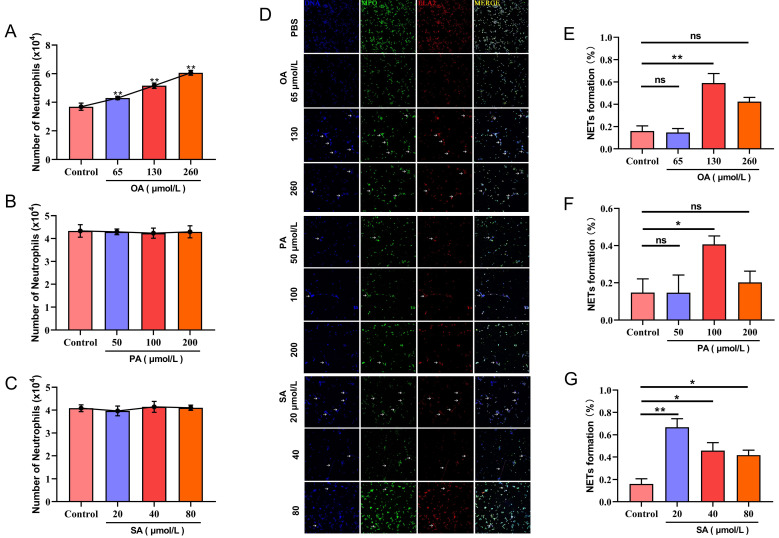
Effects of OA, PA, and SA on migration and NET formation of PMN. (**A**) Effects of OA on migration of PMN; (**B**) effects of PA on migration of PMN; (**C**) effects of SA on migration of PMN; (**D**) effects of OA, PA, and SA on NET formation of PMN (white arrows: NET); (**E**) effects of OA on the percentage of NET formation; (**F**) effects of PA on the percentage of NET formation; (**G**) effects of SA on the percentage of NET formation. Data are shown with means ± SD (*n* = 3). * *p* < 0.05, ** *p* < 0.01, compared to control group.

Compared with the control group, low, medium, and high concentrations of OA (0.065, 0.130, and 0.260 mM) significantly promoted PMN transepithelial migration (*p* < 0.01), and the higher the OA concentration, the more obvious the effect (Figure 2A). However, PA and SA had no significant effect on PMN transepithelial migration (Figure 2B,C). Compared with the control group, the medium concentration of OA (0.130 mM) significantly induced the formation of NETs (*p* < 0.01) (Figure 2D,E); PA had no significant effect on the formation of PMN NETs (Figure 2D,F); different SA concentrations could significantly induce the formation of NETs (*p* < 0.05), where a low concentration of SA showed the most significant effect (*p* < 0.01) (Figure 2D,G).

### 3.3. The Effects of Ac, Pr, and Bt on PMN Migration and NET Formation

**Figure 3:**
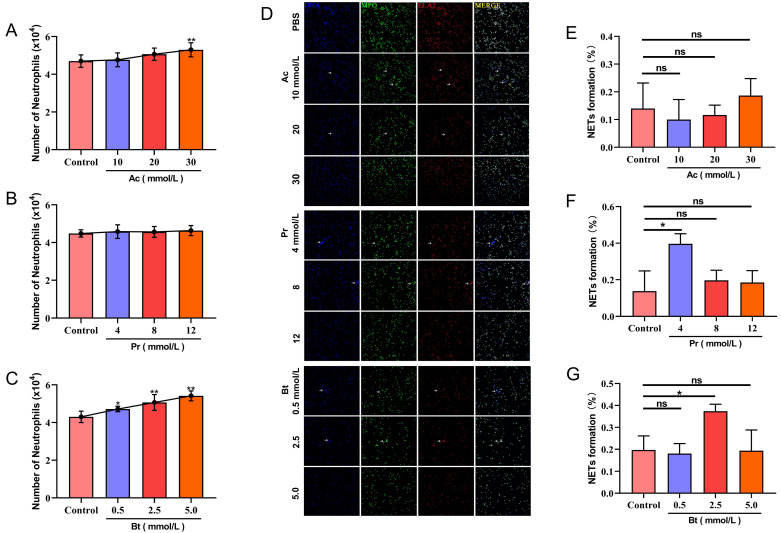
Effects of Ac, Pr, and Bt on migration and NET formation of PMN. (**A**) Effects of Ac on migration of PMN; (**B**) effects of Pr on migration of PMN; (**C**) effects of Bt on migration of PMN; (**D**) effects of Ac, Pr, and Bt on NET formation of PMN (white arrows: NET); (**E**) effects of Ac on the percentage of NET formation; (**F**) effects of Pr on the percentage of NET formation; (**G**) effects of Bt on the percentage of NET formation. Data are shown with means ± SD (*n* = 3). * *p* < 0.05, ** *p* < 0.01, compared to control group.

Compared with the control group, both a low concentration (10 mM) and medium concentration (20 mM) of Ac could promote PMN transepithelial migration and the difference was not significant (*p* < 0.05); The promoting effect of a high concentration (30 mM) of Ac could be extremely significant (*p* < 0.01) (Figure 3A). Pr had no significant effect on PMN transepithelial migration (Figure 3B). A low concentration (0.5 mM) of Bt significantly promoted PMN transepithelial migration (*p* < 0.05). A medium concentration (2.5 mM) and high concentration (5 mM) of Bt significantly promoted PMN transepithelial migration (*p* < 0.01) (Figure 3C). Compared with the control group, Ac had no significant effect on the formation of PMN NETs (Figure 3D,E). A low concentration (4 mM) of Pr significantly induced the formation of NETs (*p* < 0.05), while medium and high concentrations of Pr had no significant effect (Figure 3D,F). A medium concentration (2.5 mM) of Bt significantly induced the formation of NETs (*p* < 0.05), while low and high concentrations of Bt had no significant effect (Figure 3D,G).

## 4. Discussion

The changes in hormone secretion levels in dairy cows during the perinatal period are closely related to the immunosuppression observed in this period, which may also be one of the reasons for the increased risk of diseases for perinatal dairy cows [9]. E_2_ and P_4_ are the most important reproductive hormones in the developmental and reproductive stages of dairy cows and play an important role in pregnancy and its maintenance [20]. This study found that E_2_ in high, medium and low concentrations all inhibited the transepithelial migration of PMN, and 100 pg/mL of E_2_ significantly induced the formation of NETs. P_4_ promoted the transepithelial migration of PMN but had no obvious effect on the formation of PMN NETs. The effects of E_2_ and P_4_ on the migration ability of PMN and the formation of NETs can better explain the high incidence of diseases near parturition. Near parturition, E_2_ in the body is at the peak of secretion, and the concentration of P_4_ continues to decrease. A high concentration of E_2_ prevents PMN from entering the mammary gland acinar lumina normally to participate in the immune work. In addition, a high concentration of E_2_ can promote the production of NETs. NET was discovered to be an effective antibacterial mechanism of PMN. However, subsequent studies have shown that excessive production of NETs can damage host tissues and cause aseptic inflammation [21,22]. Therefore, NET is described as a “double-edged sword” that plays a protective and pathogenic role. Previous studies have shown that the increase of NETs can produce cytotoxic effects on bovine mammary epithelial cells (BMECs), damaging mammary tissue [23]. In conclusion, our research showed that changes in the hormone secretion levels of dairy cows during the perinatal period might cause dairy cows to be more susceptible to infectious diseases in this period.

The change in metabolites of dairy cows during the perinatal period is the other key factor causing immunosuppression. In general, the NEFA concentration in dairy cow serum is less than 0.4 mM, while that in a perinatal cow can reach 1.5 mM. Therefore, perinatal dairy cows with a high NEFA concentration (1.5 mM) may have OA, PA, and SA concentrations reaching 0.240 mM, 0.210 mM, and 0.100 mM, respectively. This study found that OA in high, medium and low concentrations all promoted transepithelial migration ability of PMN, and a medium concentration of E_2_ significantly induced the formation of NETs. PA had no significant effect on PMN transepithelial migration and NET formation. Although SA did not affect PMN transepithelial migration, it significantly induced the formation of NETs, even at a low concentration (0.02 mM). Many previous studies have shown that a high concentration of NEFAs produced by intense lipid mobilization in perinatal dairy cows significantly impaired the function of lymphocytes [24,25]. A high concentration of NEFAs can also impair PMN function. For example, NEFA can induce PMN to increase ROS production, reduce cell viability, and increase cell necrosis [9,15,16]. A recent study showed that OA induced the formation of NETs and the release of ATP through the activation of PANX1 and P2X1. PANX1 is a release channel involved in the derivation of ATP by PMN, and P2X1 is a PMN purinergic receptor [26]. In human bodies, P2X1 is involved in the migration, degranulation, and phagocytosis of PMN. Studies have shown that the activation of PMN P2X1 can promote the migration of PMN across the vascular endothelium to tissues [27]. Other studies have also shown that activating P2X1 can promote the random migration of PMN [28,29]. However, the mechanism of how SA promotes the formation of NETs still needs further research. In conclusion, our research showed that both OA and SA could promote the formation of NETs, and OA could also promote the transepithelial migration of PMN. This result supports the fact that a high concentration of NEFAs is one of the factors leading to the immune function impairment of perinatal dairy cows and postpartum diseases.

SCFA is a fatty acid composed of 2–6 carbon atoms. Acetic acid, propionic acid and butyric acid account for more than 95% of SCFA and acetic acid solely accounts for 70%. SCFA not only participates in energy metabolism and endocrine response but also regulates immune cell function [30,31,32]. The concentration of SCFAs in the rumen is much higher than that in the peripheral blood. Our results showed that under the stimulation of a physiological concentration of SCFAs in the rumen, Ac significantly promoted the transepithelial migration of PMN but did not affect the formation of NETs. Although Pr did not affect PMN transepithelial migration, it significantly induced the formation of NETs, even at a low concentration (4 mM). Bt promoted PMN transepithelial migration, and a medium concentration (2.5 mM) of Bt also significantly induced the formation of NETs. The role of SCFAs in promoting PMN migration may be achieved through two paths. In the first path, after PMN is stimulated by SCFA, the expression of L-selectin on the PMN surface is upregulated. As an adhesion molecule regulating the adhesion and rolling of PMN, L-selectin can enhance the migration of PMN. In the second path, SCFAs can bind to GPR43 on the surface of PMN, which triggers a PMN Ca^2+^ influx and actin remodeling and induces PMN morphological changes and cell polarization, thereby enhancing PMN migration ability [33,34]. There is some controversy regarding the effect of SCFAs on the formation of NETs. Ohbuchi et al. showed that Ac did not induce the formation of NETs in vitro, while Íñiguez-Gutiérrez et al. found that Ac induced the formation of NETs, even if Ac concentration was below the average blood concentration [35,36]. The results of the present study showed that with the increase of the Ac stimulation concentration, the formation rate of NETs also showed an upward trend. The possible explanation is that the pH can adjust the formation of PMN NETs, and when PMN is in an acidic environment, it prevents the formation of NETs. In addition, our results are similar to those of Carretta et al., that is, Bt can induce the formation of NETs [19]. Our research results support the fact that SCFAs are involved in regulating the migration function of PMN and the formation of NETs and thus participate in the immune regulation of perinatal dairy cows.

## 5. Conclusions

The results of this study indicate that P_4_ and Ac can regulate PMN transepithelial migration; SA and Pr can regulate PMN NET formation; E_2_, OA and Bt can regulate PMN transepithelial migration and NET formation. Our results could further explain why the changes in hormone secretion and metabolites in perinatal dairy cows are the key factors that cause immunosuppression and increased risk of disease in dairy cows

## Data Availability

Data sharing not applicable.
